# Do Systemic Factors Influence the Fate of Nonunions to Become Atrophic? A Retrospective Analysis of 162 Cases

**DOI:** 10.1155/2019/6407098

**Published:** 2019-02-19

**Authors:** Markus Rupp, Stefanie Kern, Thaqif El Khassawna, Abdullah Ismat, Deeksha Malhan, Volker Alt, Christian Heiss, Michael J. Raschke

**Affiliations:** ^1^Justus-Liebig-University Giessen, University Hospital Giessen and Marburg, Campus Giessen, Department of Trauma-, Hand- and Reconstructive Surgery, Rudolf-Buchheim-Strasse 7, 35385 Giessen, Germany; ^2^Justus-Liebig-University Giessen, Experimental Trauma Surgery, Aulweg 128, 35392 Giessen, Germany; ^3^Westfaelische-Wilhelms-University of Muenster, University Hospital Muenster, Department of Trauma-, Hand- and Reconstructive Surgery, Albert-Schweitzer-Campus 1, W1, 48149 Muenster, Germany

## Abstract

**Introduction:**

Nonunions are a challenge for orthopedic surgeons. In hypertrophic nonunions, improvement of mechanical stability usually is the satisfactory treatment, whereas in atrophic nonunions improvement of the biological environment is most important. However, scientific evidence revealed that “avital” nonunions are not avascular and fibrous tissue contains cells with osteogenic potential. To find out if systemic factors suppress this intrinsic potential in atrophic nonunions, this study compares characteristics of hypertrophic with atrophic nonunion patients.

**Methods:**

We analyzed medical records of 162 surgically treated patients suffering from aseptic long bone nonunions. Atrophic and hypertrophic nonunions were distinguished by absence or presence of callus and calcification in the fracture gap. Mechanical implant loosening and patient characteristics such as age, gender, and body mass index were assessed. Fracture classification according to AO/OTA, open and closed fractures, and osteosynthesis were recorded. In addition, comorbidities and allergies between both groups were compared.

**Results:**

A higher number of hypertrophic nonunion patients were male with often allergies. Hypertrophic nonunion occurred more often after intramedullary nailing compared to atrophic nonunions. Atrophic nonunion patients being nonallergic were significantly older than nonallergic patients suffering from hypertrophic nonunions. In both atrophic and hypertrophic nonunion patients, age was lower in patients with accompanying injuries compared with age of patients with isolated fractures.

**Conclusion:**

Systemic factors influence development of nonunion types. In nonallergic patients, atrophic nonunions occur more often in the elderly. This manuscript is a first step to identify different factors which might influence the nature of nonunion. To enable nonunion treatment which is tailored to individual patient characteristics, further prospective studies with more sophisticated research methods are necessary.

## 1. Introduction

Bone healing is a unique repair procedure which enables restoration of skeletal integrity. Orthopedic procedures like osteotomies, arthrodesis, and osteosynthesis could not be performed without this astonishing ability of the organism. Disorder of healing may result in nonunion, which is defined as cessation of bone healing after fracture of a bone. The American Food and Drug Administration (FDA) determined a minimum of 9 months to define nonunion. However, in clinical routine, nonunions usually are determined after 6 months of injury when no progressive signs of bone healing occur. Treatment of such nonunion cases is not possible without surgical intervention [1].

Among orthopedic surgeons it is well accepted that either biological or mechanical factors lead to nonunion. In 2007 the “diamond concept” was introduced to summarize all contributing reasons to bone restoration. Those are biological factors such as osteogenic cells, osteoconductive scaffolds, and growths factors as well as mechanical stability and proper vascularity [2]. Far before, Judet and Judet in 1958 and later Weber and Cech in the 1970s classified aseptic nonunions according to their radiological and biological characteristics. The brothers Judet differed between hypertrophic or hypervascularized and atrophic or avascularized nonunions. Later, Weber and Cech described hypertrophic, oligotrophic, and atrophic nonunions. They showed that hypertrophic and oligotrophic nonunions are vascular, but the atrophic ones are not. Thus the same radiological appearance would make distinction difficult [3]. Until today, however, the therapeutic approach is based on the Weber-Cech classification. Hypertrophic nonunions are treated by improvement of mechanical stability, whereas for atrophic nonunions treatment consists of debridement of fibrous tissue, reosteosynthesis, and biological stimulation by bone grafting and growth factor application [4]. Although different therapeutic approaches have been shown, evidence exists that hypertrophic and atrophic nonunions are not as different as longtime assumed. Similar macroscopical appearance as well as histological findings of fibrous and cartilaginous tissue of both types of nonunions could be shown [5–7]. Further studies have shown a cell population of fibrous tissue within both types of nonunion, which resembles bone marrow stromal cells [5, 8, 9]. In addition, no differences in alkaline phosphatase (ALP) activity and pulsed electromagnetic field stimulation in cultures from atrophic and hypertrophic nonunions were detected [10]. Reed and others could not find a difference between atrophic and hypertrophic human nonunion tissue on vascularity [6].

This study was aimed at identifying systemic factors that influence the nature of nonunion fractures. This retrospective analysis focused on patient, injury, and treatment characteristics against the known nonunion development risk factors [11] and recent insights in “osteoimmunology” [12]. Special attention was paid to allergies. The allergies were recently described as protective for nonunion development in an epidemiological study [13]. Also, it represents both a clinical immunological as well as easy to assess patient characteristic. To the best of our knowledge, this is the first clinical study comparing risk factors that are conceivably relevant for either hypertrophic or atrophic development of aseptic nonunions.

## 2. Methods

The study was approved by the local ethical committee. We analyzed data of all surgically treated patients suffering from nonunion between January 2005 and December 2010. All included patients aged 18 years or older. Nonunion of long bones such as femur, tibia, fibula, humerus, radius, ulna, and clavicle were noticed. Nonunion was defined as permanent end of bone healing for more than 6 months. Patients with delayed healing for less than 6 months were excluded of the study. Further, patients treated for septic nonunion were not involved for the study. Septic nonunion was diagnosed by presence of a sinus tract, purulent discharge, exposed osteosynthesis implants, and a positive “probe to implant” test. Besides, a positive culture result of microbiological testing and a histologically confirmed infection (>5 granulocytes in a field of view at a magnification of 400) were reliable diagnostic features in septic nonunion. More than 2000 leucocytes/*μ*l in synovial fluid or >70% granulocytes of cells in synovial fluid of accompanying septic arthritis were considered to confirm septic nonunion.

Patient characteristics such as age, gender, body mass index (BMI; calculated as weight in kilograms divided by height in meters squared) were analyzed. Evaluation of obesity and possible link to nonunion was assessed in this study. People were considered obese with a BMI over 30 kg/m^2^. The range of 25-30 kg/m^2^ was defined as overweight, 18.5-25 kg/m^2^ as normal weight, and <18.5 kg/m^2^ as underweight. Accompanying injuries as possible influence factors of nonunion development were analyzed as well. Common risk factors for nonunion development such nicotine abuse, cardiovascular diseases, and diabetes mellitus were assessed for all patients. Cardiovascular disease included coronary artery disease, peripheral artery disease, and history of myocardial infarction or stroke. In addition, medical records were searched for atopic dispositions as well as known allergies. Nonsteroidal anti-inflammatory drugs (NSAIDs) which are known to influence bone healing were assessed as well. Permanent NSAID intake until revision surgery was compared to none or pro re nata (PRN) medication.

The long bones were numbered as follows according to the AO or OTA fracture classification: 1=humerus, 2=forearm, 3=femur, and 4=lower leg. The second number shows that the fracture position and numbers were assigned with 1=proximally, 2=diaphyseal, 3=distally, and 4=ankle. The clavicle was numbered as 15.

**Figure 1:**
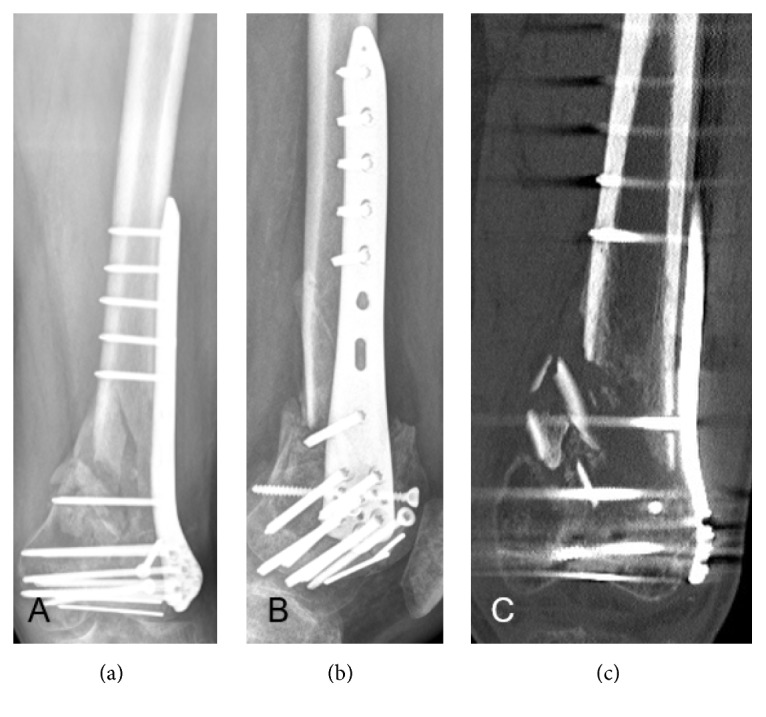
Nonunion 9 months after metaphyseal fracture of the distal femur. Neither X-rays ((a)=anterior-posterior; (b)=lateral) nor CT showed callus formation around the cortices and calcification of the fracture within the fracture zone. Thus, nonunion was considered atrophic.

Fractures were distinguished between open and closed fractures. Nonunions were assigned as hypertrophic and atrophic. Conventional X-rays in two planes and CT scans were evaluated. An atrophic nonunion was diagnosed when no callus around the fracture gap and no signs of bony consolidation within in the fracture gap were obvious. To classify a nonunion atrophic, on both radiological planes no signs of cortical bridging by fracture callus as well as no calcification within the fracture gap were allowed to be present (Figure 1).

**Figure 2:**
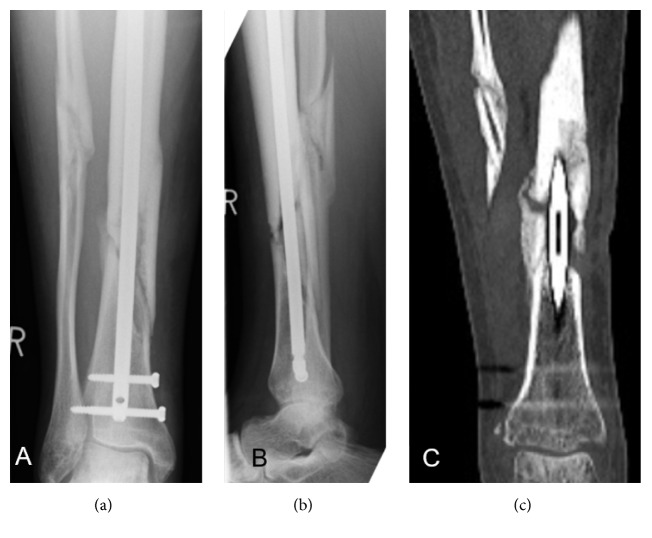
Nonunion 8 months after diaphyseal fracture of the tibia after intramedullary nailing. According to anterior-posterior (a) and lateral (b) view, classification of nonunion as hypertrophic or atrophic was difficult. CT (c) revealed callus formation and incomplete bridging of the lateral cortex, which led to classify this nonunion hypertrophic.

If performed before revision surgery, all 3 section levels (sagittal, coronar, and transverse) of CT images were evaluated for callus formation and calcification of the fracture gap as well. If callus formation and signs of at least partial consolidation were present, nonunions were considered to be hypertrophic (Figure 2).

As possible reason for nonunion development implant loosening was assessed by X-rays taken before revision surgery. We focused on clear and reproducible radiological signs: plate, screw, and wire breakage as well as lysis zones around implants as sign of implant movement. Conceivably mechanical reasons, such as too small implants, that is small diameters of intramedullary nails or possibly too thin plates, were not considered. However, fracture fixation was evaluated. We distinguished between main fracture fixation procedures such as intramedullary nailing, plating, screw fixation and wire cerclage fixation. If a combination of osteosynthesis procedures was performed, the main osteosynthesis proceedings were chosen. For example, if intramedullary nailing with added wire cerclages was performed, osteosynthesis was considered to be intramedullary nailing. Laboratory tests before nonunion revision surgery provided information of leukocytes (thousands/*μ*l) and c-reactive protein as inflammatory markers. Time between osteosynthesis of the fracture and nonunion revision surgery was determined as well.

Data were analyzed using SPSS statistics version 24.0 (IBM, SPSS Inc., Armonk, NY). Frequencies for all nonunion risk factors were calculated. For analyses of differences between patients, the chi-squared test (*X*^2^ test) or Fischer's exact test was applied for categorical variables. Mann-Whitney U-test was applied for intergroup and intragroup comparisons. The critical value for significance was set at p<0.05. Data were presented in graphs as means ± standard error of the mean (SEM). Odds ratio and confidence interval (CI) for the different parameter and their effect on atrophic and hypertrophic nonunion were calculated using 2x2 contingency table in chi-square test using SPSS statistics version 24.0.

## 3. Results

### 3.1. Patient Characteristics

In total, 206 patients were treated for long bone nonunion in the set time frame. Out of 206 patients, 36 were excluded because of the diagnosis of septic nonunion. Also, 8 patients were excluded because of age < 18 years. Therefore, 162 patients remained in the study.

**Figure 3:**
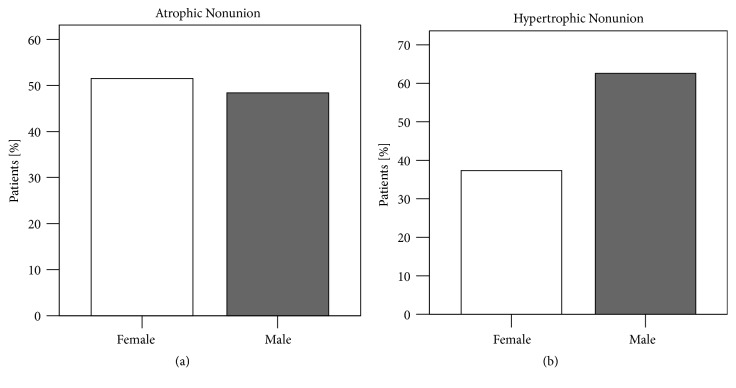
Frequencies of (a) atrophic and (b) hypertrophic nonunions depended on patient's gender. Hypertrophic nonunion patients were male in 62.2% while numbers of male and female patients with atrophic nonunions were almost even with 52.4% female versus 47.6% male patients suffering from atrophic nonunions. However, no significant association was found *X*^2^(1)> = 3.534, p=0.074) (*X*^2^ test, *∗*= p <0.05).

Overall, 92 (56.8 %) patients were men and 70 (43.2 %) women. The average age was 51.30 ± 16.34 years (ranging from 19 to 88 years). Of 162 participants in the study, 99 patients (61.1%) suffered from a hypertrophic nonunion, while 63 patients (38.9%) had an atrophic nonunion. Hypertrophic nonunion patients were more often male, 62 (62.2%). Thirty-seven (37.4%) of the hypertrophic nonunion patients were female. In atrophic nonunions, numbers of male and female patients were almost even with 33 (52.4%) female and 30 male patients (47.6%). However, no association was found between gender and type of nonunion *X*^2^(1)> = 3.534, p=0.074) (Figure 3 and Table 1).

### 3.2. Fracture and Fixation Characteristics

Classification of fracture localization after AO/OTA showed differences between atrophic and hypertrophic nonunions. In both types, nonunions were more prominent in femur fractures (n=20, 31.7% of atrophic nonunions; n=33, 34.0% of hypertrophic nonunions). By comparing atrophic with hypertrophic nonunions, data showed that atrophic nonunions occurred more in the forearms (n=19, 30.2%) compared with hypertrophic nonunions (n=16, 16.5%). Percentage of hypertrophic nonunions of tibial fractures (n=23, 23.7%) was higher compared with atrophic nonunions (n=12, 19.0%). There were no differences seen in humerus fractures between both nonunion types (atrophic: n=10, 15.9%; hypertrophic: n=15, 15.5%). Patients with clavicle fracture had more hypertrophic nonunions (n=10, 10.3%) compared with atrophic nonunions (n=2, 3.2%).

**Figure 4:**
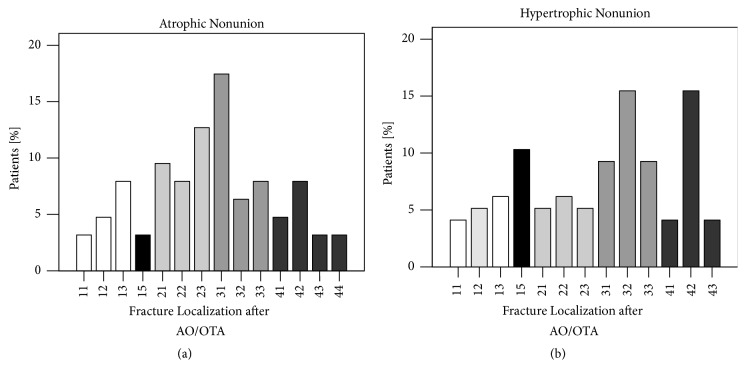
Atrophic and hypertrophic nonunion patients exhibited different fracture localization. Number XY: X=1=humerus, 2=forearm, 3=femur, and 4=lower leg. Y=1=proximally, 2=diaphyseal, 3=distally, and 4=Ankle. 15=Clavicle. No association was found between nonunion type and fracture localization after AO/OTA (*X*^2^(15)> = 18.533, p=0.236) (*X*^2^ test; *∗*= p <0.05).

Hypertrophic nonunion occurred more often in diaphyseal long bone fractures (n=41, 42.3%) compared to atrophic nonunion (n=17, 27.0%) (Figure 4). However, no significant association was found between fracture localization and type of nonunion (*X*^2^(15)> = 18.533, p=0.236).

**Figure 5:**
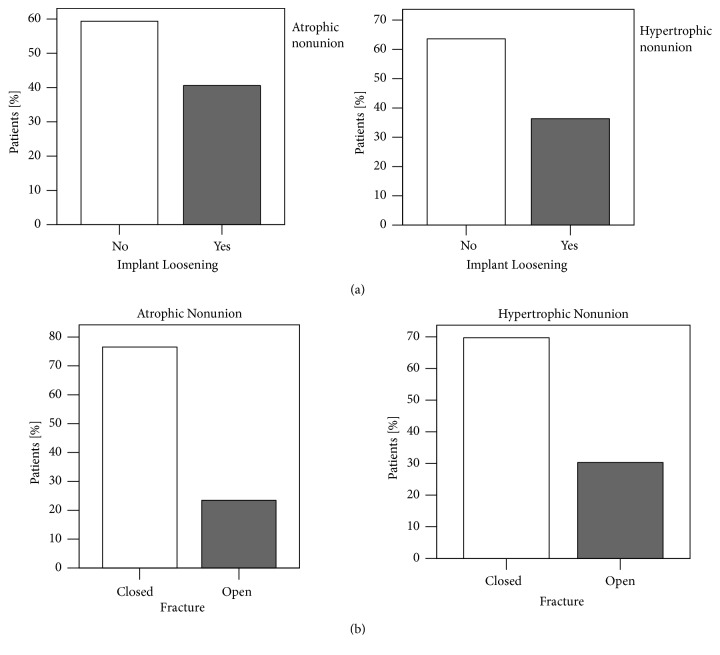
Frequency analysis of fracture-implant characteristics. (a) Radiological evident implant loosening occurred more often in atrophic nonunion (n=26, 41.3%) compared with hypertrophic nonunion patients (n=36, 36.4%). However, no association was found between nonunion type and implant loosening (*X*^2^(1)> = 0.392, p=0.619). (b) Frequency of open fractures leading to nonunion was higher in the hypertrophic (n=30, 30.3%) compared to the atrophic group (n=14, 22.2%). There was also no association between fracture type and nonunion type (*X*^2^(1)> = 1.271, p=0.282) (*X*^2^ test, *∗*= p <0.05).

Comparing hypertrophic with atrophic nonunions, no statistically significant differences of implant loosening could be noted (*X*^2^(1)> = 0.392, p=0.619) (Figure 5(a) and Table 1). 36.4% of hypertrophic nonunions showed radiological evidence of implant loosening, whereas 41.3% of atrophic nonunions had a diagnosed loosening of their implant. Frequencies of implant loosening were higher in female compared to male patients for both atrophic and hypertrophic nonunions cases, not statistically significant though. In 27.2% (n=44) open fractures led to nonunion, whereas in 72.8% (n=118) closed fractures resulted in nonunion development (Figure 5(b) and Table 1). No difference was found comparing nonunion type in this group (*X*^2^(1)> = 1.271, p=0.282).

Overall, there were more patients suffering from nonunions treated with plates (n=81; 50.0 %) than with intramedullary nails (n=55; 34.0%) or screw fixation (n=20; 12.3%) of the fracture. Nonunion resulting from fractures treated with cerclage showed the lowest percentage (n=6; 3.7%).

**Figure 6:**
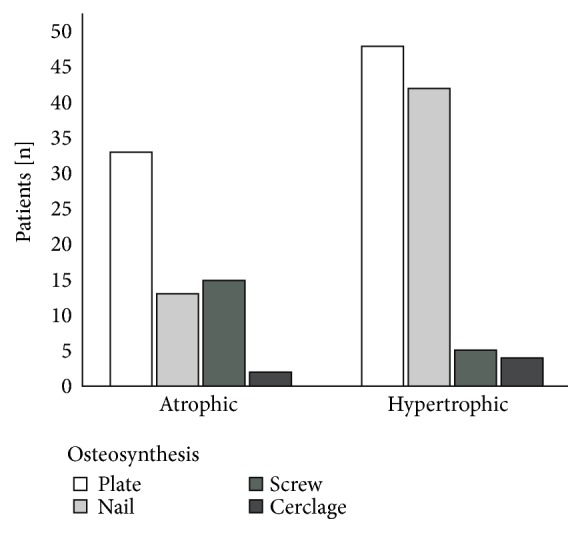
Influence of osteosynthesis material on nonunion type. Hypertrophic nonunion occurred more often after intramedullary nailing compared to atrophic nonunions. Fracture treatment with screws more often led to atrophic nonunions compared to hypertrophic nonunions (*X*^2^(3)> = 16.553, p=0.001) (*X*^2^ test, *∗*= p <0.05).

Nonunion type was associated with osteosynthesis material (*X*^2^(3)> = 16.553, p=0.001). In hypertrophic nonunions intramedullary nailing was more often than in atrophic nonunions (hypertrophic: n=42, 42.4%; atrophic: n=13, 20.6%). For screw fixation, it was conversely with 15 patients (23.8%) in the atrophic nonunion group and 5 patients (5.1%) in the hypertrophic nonunion group (Figure 6).

### 3.3. Comorbidities and NSAIDs

Comparing patient comorbidities and medication of NSAID no significant differences between patients suffering from atrophic and hypertrophic nonunions were found. Nonetheless, more than 40% patients in both groups suffered from at least one of the comorbidities which were evaluated in this study (nicotine abuse, cardiovascular diseases, and diabetes mellitus). Differences in type and numbers of the assessed comorbidities were not different between atrophic and hypertrophic nonunion patients (*X*^2^(6)> = 4.970, p=0.573). Permanent NSAID intake was more frequent in hypertrophic nonunion patients (20.3% for hypertrophic versus 27.3% in atrophic nonunions). However, no significant difference could be determined (*X*^2^(1)> = 0.912, p=0.358) (Table 1).

### 3.4. Obesity

In our study, 58 patients (36.0%) were normal weighted. Two-thirds of participants in this study had a BMI over 25. In total 100 patients (62.1%) had overweight, divided into 44 obese patients (27.3%) and 56 overweighed patients (34.8%). Only 3 of 162 patients (1.9%) were underweighted. There was no association with type of nonunion and BMI (*X*^2^(3)> = 1.878, p=0.636).

**Figure 7:**
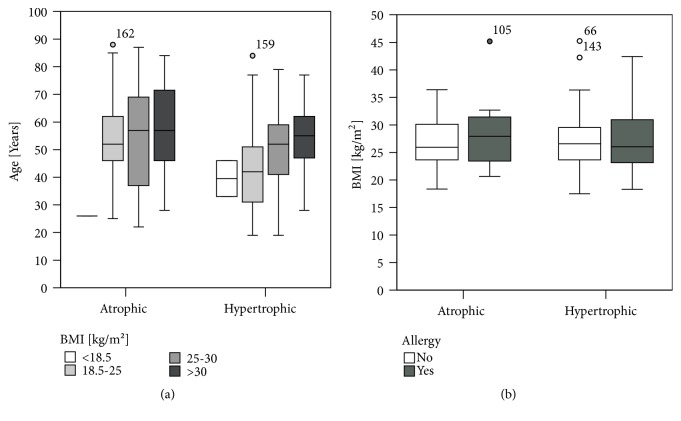
Obesity correlates with age in nonunion patients. (a) Body mass index increased with age in hypertrophic as well as atrophic nonunion patients. (b) Atrophic nonunion patients had higher BMI than nonallergic patients, although not statistically significant (Mann-Whitney U-test, *∗*=p≤0.05).

BMI increased with increasing age in both patient groups, atrophic and hypertrophic. Allergic patients with an atrophic nonunion had a higher BMI than nonallergic patients, although not significantly (Figure 7).

### 3.5. Allergies

An association was found in patients with allergies and type of nonunion (*X*^2^(1)> = 4.311, p=0.041). Patients who needed revision surgery for hypertrophic nonunions had an allergy in more than 30.3% (n=30), atrophic nonunion patients in less than 15.9% (n=10).

**Figure 8:**
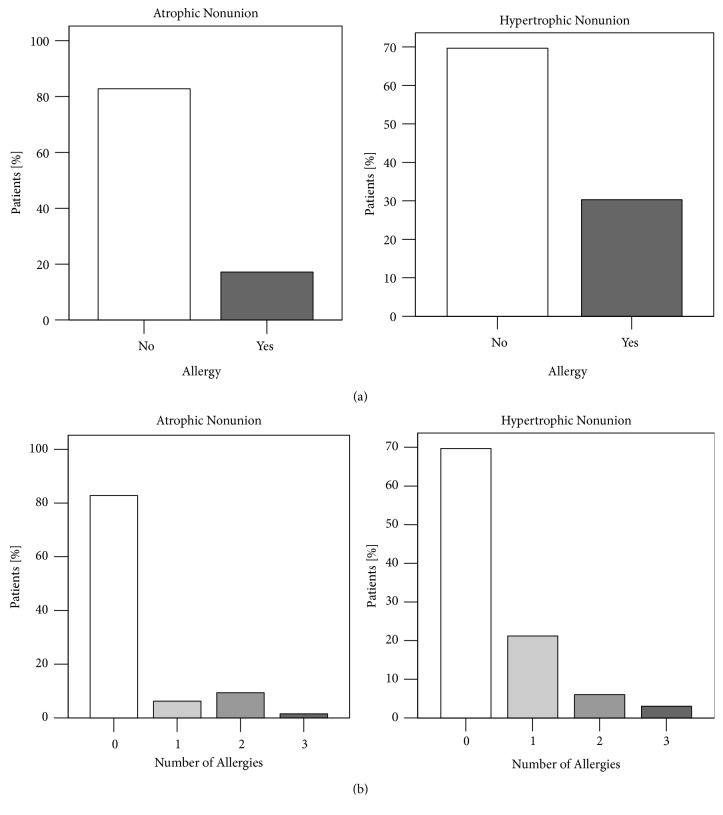
Frequency analysis of allergies in nonunion patients. (a) Hypertrophic nonunion patients suffered at least one allergy in 30.3% (n=30) whereas 15.9% (n=10) of the atrophic nonunion patients were allergy sufferers. An association was found between allergies and type of nonunion (*X*^2^(1)> = 4.311, p=0.041), patients with allergies were more prone to hypertrophic nonunions. (b) No difference significant difference in numbers of allergies could be evidenced comparing atrophic with hypertrophic patients (*X*^2^(3)> = 7.100, p=0.069) (*X*^2^ test, *∗*= p <0.05).

The allergies assessed in this study were food allergy (1.75% of all allergies), 9 pollen allergies (15.19%), 4 allergies against metals (7.02%), 3 insecticide allergies (5.26%), 25 allergies against drugs (43.86%), 1 allergy against contrast agent (1.75%), and 14 contact allergies (24.56%). Frequency analysis showed that patients with allergies had mostly one allergy followed by two and three allergies. No differences in types and number between the atrophic and hypertrophic group could be determined (*X*^2^(3)> = 7.100, p=0.069) (Figure 8 and Table 1).

**Figure 9:**
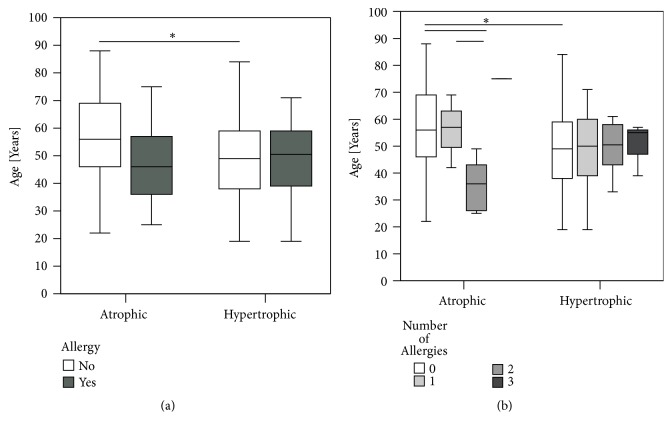
Age of patients differed with numbers of allergies. (a) Patients without allergies suffering atrophic nonunions were significantly older than patients with hypertrophic nonunion (p=0.020). (b) Patients with atrophic nonunion and two allergies were significantly younger than atrophic nonunion patients with none (p=0.0149) or only one allergy (p=0.049) (Mann-Whitney U-test, *∗*=p≤0.05).

Significant difference between the ages of patients with no allergy was seen in the nonunion. Patients with an atrophic nonunion were older compared to patients with hypertrophic nonunion (Mann-Whitney U-test, p=0.020). The study also revealed significant differences in the age of patients with atrophic nonunion. Patients with two allergies were significantly younger than patients with none (Mann-Whitney U-test, p=0.0149) or one allergy (Mann-Whitney U-test, p=0.049) (Figure 9).

BMI of patients with either atrophic or hypertrophic nonunion was by trend increasing with increased number of allergies. The duration between surgery for definitive fracture osteosynthesis and nonunion revision was highest in patients with three allergies in hypertrophic nonunion patients. Further, there was an inhomogeneity in patients with atrophic nonunion with the longest duration in patients with two allergies.

### 3.6. Concomitant Injuries

**Figure 10:**
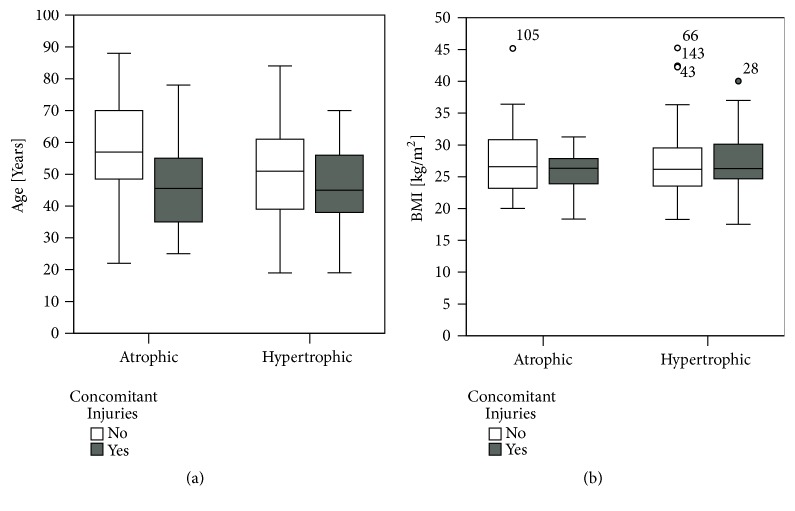
Concomitant injuries appeared more often in younger nonunion patients. (a) Although no significant difference was observed, data evaluation showed younger age of patients with accompanying injuries compared to monofracture patients for both atrophic and hypertrophic nonunion patients. (b) No statistically significant difference of body mass index could be evidenced comparing atrophic and hypertrophic nonunion patients with concomitant injuries. However, BMI of patients with atrophic nonunion and concomitant injuries was lower compared to patients with only one injury. In hypertrophic nonunion patients, the contrary could be evidenced (Mann-Whitney U-test, *∗*=p≤0.05).

Overall, 53 (32.7 %) of all 162 patients showed concomitant injuries, which made additional fracture fixation necessary. No association was found between nonunion type (atrophic or hypertrophic) and concomitant injuries (*X*^2^(1)> = 2.509, p=0.113) (Table 1). Of 63 patients with atrophic nonunion, 16 (25.4%) had concomitant injuries. In the group of hypertrophic nonunion patients (n=99), 37 (37.4%) had concomitant injuries. Interestingly, nonunion patients with concomitant injuries were younger compared to patients with only one fracture. This was seen in both atrophic and hypertrophic nonunion patients (Figure 10(a)).

No statistically significant differences were noted comparing BMI of atrophic and hypertrophic nonunion patients with no or present accompanying injuries (Figure 10(b)).

## 4. Discussion

Evidence of an intrinsic ability of bone healing in atrophic nonunions is a positive aspect, especially for recalcitrant nonunion treatment. In future, knowledge about systemic factors influencing nonunion development may provide insights to improve the biological microenvironment by medical instead of surgical treatment alternatives.

Gender influence was compared in atrophic and hypertrophic nonunion patients, to examine the effect of sexual hormones on bone metabolism. Hypertrophic nonunions were seen more in male patients than female patients. However, the difference between the number of males in atrophic and hypertrophic group was not statistically significant (Figure 3). Experimental data comparing bone healing in male and female rats could display a compromised callus formation and delayed callus bridging for female rats. Fewer mesenchymal stem cells (MSCs) were found at the fracture site as well, while characteristics of MSCs were the same [14]. The occurrence of more hypertrophic nonunions among men is in accordance with those findings.

For open fractures, the frequencies of hypertrophic and atrophic nonunions are not significantly different. As assumed, nonunions occurred often after open fracture. About 27% of all aseptic nonunions were seen after open fractures. In literature, open fractures account for 2.6% of all fractures [14]. Thus, the high risk of nonunion development is reflected by the present data. Also, the present study analyzed only aseptic nonunions. Septic nonunions, which represent a significant part of nonunions after open fracture [15], have not been included into the study. However, aseptic nonunions could be expected to be found more often after open fractures, since pronounced soft tissue damage in open fractures was considered to weaken the biological fracture environment. Nonetheless, more hypertrophic nonunions after open fractures, but no significant difference in the frequencies of atrophic and hypertrophic nonunions could be shown (Figure 5(b)).

Impaired mechanical stability is widely accepted to be reason for nonunion development. The current analysis revealed no difference between frequencies in hypertrophic and atrophic nonunions related to radiographic signs such as breakage of implants, loosening zones around implants, and screw migration (Figure 5(a)). Local strain within the fracture is not accessible by radiographic evaluation alone. This might explain why a higher but not statistically significant rate of atrophic nonunions was seen with implant loosening. Tools such as finite element might enable better assessment of nonunions' mechanics in future clinical studies [16]. Nonunions resulting after intramedullary nailing were more often hypertrophic while more atrophic nonunions occurred after plate or screw osteosynthesis (Figure 6). Although the differences between fixation techniques for atrophic and hypertrophic nonunions were not statistically significant, findings were consistent with the accepted doctrine for bone healing. On the one hand, rigid fixation avoids callus formation. Furthermore, anatomic reduction and consecutive direct bone healing result in less callus development. On the other hand, indirect bone healing after dynamic osteosynthesis performed with intramedullary nails results in callus formation [17].

Zura et al. found that 19.5% of all patients treated for fractures also suffered from an allergy. They could find few nonunions in course of the treatment compared to nonallergic patients [13]. This data analysis of a large payer database included diagnosed allergies based on clinical information. In the current study, 25.2% of the patients suffered from an allergy or allergic disposition based on clinical diagnosis as well (Figure 8). The reported prevalence of allergies in patients of Western countries is similar (i.e., 28.1% in Germany in 2016) [18]. Comparing atrophic to hypertrophic nonunions, more patients with hypertrophic nonunions had at least one allergy ((*X*^2^(1)> = 4.311, p=0.041). Emerging knowledge about the critical role of the innate as well as adaptive immune system in fracture healing is coined by the term “osteoimmunology” [19]. Not only during the inflammatory phase of bone healing, but also for the repair [20] and remodeling phase [21] immune cells play a critical regulative role. Promising experimental data has been recently published showing improved fracture healing by modulating peripheral blood mononuclear cells in an immunodeficient rat femur nonunion model [22]. The protective effect of allergies in bone healing described by Zura and coworkers still has to be unfolded [13]. Clinical results from present study could not explain this phenomenon. Further, they cannot answer the question how altered immune system in allergies affect fracture healing.

Age is known to be a risk factor for nonunion. Besides age-related changes of macrophages, T cells and MSCs, vascularization and angiogenesis are impaired in bone healing in the elderly. Further, bone cells and their progenitors show a lower activity and a smaller number within callus [23]. The present study determined that patients without allergy suffering from an atrophic nonunion were significantly older than nonallergic patients with hypertrophic nonunion. Increased prevalence of allergies in younger patients could explain this difference [24].

Hitherto, success of fracture reduction and internal fixation with metallic implants did not rise the question of metallic implants themselves being a possible reason for impaired fracture healing. However, in cases without obvious cause for nonunion development, the metallic implant might be the reason for failure in fracture healing. In arthroplasty, severe inflammation in response to wear debris and metal ions is the leading cause of local osteolysis with consecutive implant failure [25]. The immune response to wear debris and metal ions is complex and not fully understood. However, the general understanding is that wear debris lead to macrophage activation. Released chemokines like monocyte chemoattractant protein-1 (MCP-1), macrophage inflammatory protein-1 alpha (MIP-1a), and interleukin 8 (IL-8) draw other macrophages and osteoclasts to the site of inflammation. Cytokines like interleukin-1*β* (IL-1*β*), tumor necrosis factor *α* (TNF-*α*), interleukin-6 (IL-6), and prostaglandin E2 (PGE2) were released. Higher levels of receptor activator of NF-*κ*B ligand (RANKL) result in increased osteoclast activity and bone resorption [26]. Further, T lymphocyte (type IV, delayed hypersensitivity) mediated immune reactions to metal ions of cobalt, chromium, molybdenum, and titanium have been determined. Those metals make up the most part of alloys of currently used orthopedic implants [25]. It would be a new approach to compare aseptic loosening as a longtime sequela after prosthesis implantation to disturbance in the relatively short periods of fracture healing. Nonunion rates in operated patients are higher than in conservatively treated patients. However, it is obvious that several other reasons beside hypersensitivity to the osteosynthesis implant could be accountable for higher nonunion rates after surgical treatment [13]. Also, allergy rates similar to the rates in the Western population do not underpin the implant allergy hypothesis. Nevertheless, since the key players of aseptic loosening are the same for fracture healing, implant related failure of fracture healing should be considered, at least in recalcitrant nonunions. Future insights of the interplay between bone metabolism and immune system might elucidate if implants are negative factors for fracture healing and how implants should be improved to influence bone healing in the needed direction.

There was no statistically significant difference between hypertrophic and atrophic nonunions in patients treated for one compared to two or more fractures, which needed surgical care (Figure 10). Until today, clinical studies, which describe the impact of additional fractures on bone healing, are lacking. For multiple trauma the risk of delayed union or nonunion is higher [13]. However, increased callus formation and heterotopic ossification could be demonstrated in extensive trauma including traumatic brain and spinal cord injury [27, 28]. Although the complex interplay between cells, hormones and cytokines in fracture healing is by far not well understood, concomitant traumatic brain and spinal cord injury result in a serum leptin increase which is deemed responsible for improved callus formation [29, 30]. This suggests fracture healing and callus formation being dependent on the concomitant injury pattern. Soft tissue injury, hemorrhage, and chest trauma could result in local and systemic hypoxia. Both could lead to impaired fracture healing [31]. Additional soft tissue trauma has not influenced bone healing in rats [32], whereas further chest trauma has led to impaired fracture healing in a rat animal model [33]. To what extent hemorrhage influences fracture healing is not clear, yet. While hemorrhagic shock retards fracture healing during the early phase of the fracture healing process in an experimental mouse model [34], others could not evidence any difference in fracture healing of goats with and without additional hemorrhagic shock [35]. Nonetheless, larger callus with evidence of delayed bone remodeling could be observed in a rabbit model examining the impact of hemorrhage on fracture healing [36]. The observation period, fracture stabilization, and method of hemorrhagic shock in different kinds of animal models could be reason for those divergent findings. It needs to be clarified by further clinical studies, if the high rate of nonunion patients with concomitant fractures is based on systemic reasons such as hemorrhage, chest trauma, soft tissue injury, or simply additional fracture. Systemic metabolic, inflammatory, or neuroendocrine mediators, which could be responsible for impaired fracture healing, would be a possible target for medical treatment of disturbed fracture healing.

The shortcoming of the present study is its retrospective design with related drawbacks. The file review impeded a more detailed classification of tissue damage, description of comorbidities and confirmation of patient declared allergies. Especially for allergies, intolerance and side effects are often misunderstood as allergy. An exact delimitation is difficult, not only for medical laypersons. Regarding the X-ray evaluation of nonunions, only obvious mechanical reasons for nonunion development were assessed. For clinical studies, assessment of stability and flexibility is not possible. Therefore, we try to avoid interpretation failures by clearly definable mechanical criteria. Also, hypertrophic and atrophic nonunions were distinguished by evaluating of fracture gaps and callus formation on conventional X-rays and CT. However, a distinction between atrophic and oligotrophic nonunions was difficult. Oligotrophic nonunions share the biological capacity of hypertrophic nonunions but show little to no callus formation. Therefore, frequency of atrophic nonunions could be mistakenly too high. This problem could be solved in prospective studies using nuclear imaging studies for assessing biological activity [37]. Lastly, the study does not present a comprehensive analysis of systemic factors such as further secondary diagnosis and medication because of those secondary diagnoses. Because of the retrospective design no laboratory analysis for evaluation of blood cells and serum markers could be performed. Future clinical trials should focus on this essential examination.

## 5. Conclusion

Treatment of aseptic hypertrophic and atrophic nonunions according to the clinically Weber-Cech classification follows different strategies. However, evidence of an intrinsic healing potential in atrophic nonunion which might be inhibited by systemic factors has been reported in experimental studies. Only in nonallergic elderly a significant higher rate of atrophic nonunions could be determined compared to more hypertrophic nonunions in nonallergic younger patients. Further relevant systemic biological factors could not be determined in our study. Nevertheless, unfolding those factors might change current treatment principles and could open the gate not only to at least adjuvant medical but also to customized nonunion and fracture treatment. For this purpose, future prospective clinical trials with more sophisticated diagnostic tools are necessary.

## Figures and Tables

**Table 1:** Summary of events relative to atrophic versus hypertrophic nonunions depicted as odds ratio and confidence interval. If odds ratio is higher than 1, the event with the first odds is more likely in atrophic nonunions. In case of odds ratio less than 1 the second event is more likely in hypertrophic nonunions.

Parameter		Patients [n]		95 % Confidence Interval		Odds Ratio
		atrophic	hypertrophic	Lower	Upper	
allergy	No	53	69	1.035	5.130	2.304
	Yes	10	30			

gender	Female	33	37	0.971	3.497	1.843
	Male	30	62			

concomitant injuries	No	47	62	0.872	3.524	1.753
	Yes	16	37			

fracture type	Closed	49	69	0.732	3.166	1.522
	Open	14	30			

NSAID	No	50	72	0.679	3.064	1.442
	Yes	13	27			

implant loosening	No	37	63	0.426	1.554	0.813
	Yes	26	36			

## Data Availability

The data used to support the findings of this study are included within the article.
